# B Cells Contribute to Heterogeneity of IL-17 Producing Cells in Rheumatoid Arthritis and Healthy Controls

**DOI:** 10.1371/journal.pone.0082580

**Published:** 2013-12-05

**Authors:** Paul Martin Schlegel, Ingeborg Steiert, Ina Kötter, Claudia A. Müller

**Affiliations:** 1 Section for Transplantation Immunology and Immunohematology, Department II, Medical Clinic, University Hospital, Tuebingen, Germany; 2 Department of Anesthesiology and Intensive Care Medicine, University Hospital, Tuebingen, Germany; 3 Department II, Medical Clinic, University Hospital, Tuebingen, Germany; University of Tübingen, Germany

## Abstract

Secretion of the proinflammatory cytokine Interleukin-17A (IL-17A) is the hallmark of a unique lineage of CD4 T cells designated Th17 cells, which may play a crucial role in the pathogenesis of rheumatoid arthritis (RA) and many autoimmune diseases. Recently, IL-17-producing cells other than T cells have been described, including diverse innate immune cells. Here, we show that the cellular sources of IL-17A in RA include a significant number of non-T cells. Multicolour fluorescence analysis of IL-17-expressing peripheral blood mononuclear cells (PBMC) revealed larger proportions of IL-17^+^CD3^-^ non-T cells in RA patients than in healthy controls (constitutive, 13.6% vs. 8.4%, and after stimulation with PMA/ionomycin 17.4% vs. 7.9% p < 0.001 in both cases). The source of IL-17 included CD3^-^CD56^+^ NK cells, CD3^-^CD14^+^ myeloid cells as well as the expected CD3^+^CD4^+^ Th17 cells and surprisingly a substantial number of CD3^-^CD19^+^ B cells. The presence of IL-17A-expressing B cells was confirmed by specific PCR of peripheral MACS-sorted CD19^+^ B cells, as well as by the analysis of different EBV-transformed B cell lines. Here we report for the first time that in addition to Th17 cells and different innate immune cells B cells also contribute to the IL-17A found in RA patients and healthy controls.

## Introduction

Since its first description in 1993 [1], IL-17A (also referred to as IL-17) has received much attention as an important proinflammatory cytokine with a critical role in immune defence against extracellular pathogens as well as in the pathogenesis of different autoimmune diseases. It was first isolated from a cytotoxic T cell hybridoma (CTLA8) and later recognized to belong to a cytokine family which includes five additional members IL-17B, IL-17C, IL-17D, IL-17E (also known as IL-25) and IL-17F. IL-17A and IL-17F share the highest sequence homology and signal through a heterodimeric IL-17 receptor complex which comprises the two subunits IL-17RA and IL-17RC [2]. Members of this cytokine family, especially IL-17A, act in different arms of the adaptive immune response [3], as well as in the coordinated regulation of innate immunity against bacterial and fungal infections [4].

IL-17A was first described to be a signature cytokine of a new CD4^+^ T cell subset designated Th17 [5,6] which expresses the lineage-specific transcription factor retinoic acid receptor-related orphan receptor-γt (ROR γt ) and is distinct from the Th1 and Th2 subsets [7]. Differentiation of Th17 cells from naïve T cells in vivo was shown to require the cytokines IL-6 and transforming growth factor β [8-10]. Recently, it has been recognized that several other RORγt-expressing lymphocytes also secrete IL-17. In mice and/or humans, these include CD8^+^ αβ T cells [11], γδ T cells[12], LTi-like innate lymphoid cells (ILCs)[13], natural killer T cells (NKT) [14], and CD3^+^ invariant natural killer cells [15]. In addition, it is more and more accepted that diverse innate myeloid immune cells are able to produce IL-17. This has been reported for monocytes and macrophages in gut tissues of patients with Crohn´s disease and ulcerative colitis [16], for neutrophils in systemic vasculitis [17], for mast cells in psoriatic skin lesions [18]. Most recently also B cells in mice and humans have been shwon to produce IL-17 in response to infection with Trypanosoma cruzi [19].

It has also been suggested that IL-17 plays a key role in the pathogenesis of RA. Transgenic animal models provided first evidence that overexpression of IL-17 could lead to arthritis through the induction of chronic inflammation, cartilage and bone erosion in joints [20]. In rodents, it was also shown that IL-17 is present at sites of the inflamed joints and that Th17 cells represent a dominant cell type among other T cells involved in the pathogenesis of chronic erosive disease [21]. In patients with RA, exposure of synovium explants to IL-17 in vitro was demonstrated to induce molecular mechanisms of joint destruction [22]. However, conflicting results were reported on the level of IL-17 in patients' serum, synovial membranes and synovial fluid as well as on the frequency of Th17 cells in blood and inflamed tissues. Whereas several investigators reported that IL-17 levels in synovial fluids of early RA were higher than in serum [23-26], there are conflicting data on the cellular source of IL-17 in the literature [27-30]. Some authors [31,32] detected raised Th17 levels in PBMC in comparison to healthy controls, while Janduns et al. [33] found increased frequencies of Th17 cells only in patients with seronegative spondyloarthritis, but not in RA. Hueber et al. [30] reported that only 1-8% of IL-17^+^ cells were CD3^+^ T cells in synovial tissues. The same authors showed that mast cells in synovial tissues of patients with RA also express IL-17A and could substantially contribute to proinflammatory immune reactions in joints.

As mast cells belong to a heterogeneous group of innate immune cells which can produce IL-17, RA patients were further investigated for the frequency and phenotype of IL-17^+^ non-T cells in PBMC and compared to healthy controls in the present study. We show that, although the frequencies of Th17 cells in PBMC of RA patients were not significantly different from controls, there were significantly higher numbers of IL-17^+^ non-T cells in RA patients. These non-T cells were especially enriched in B cells, but also included NK cells and monocytes. Furthermore this study shows for the first time that B cells among the heterogeneous populations of IL-17^+^ non-T cells are also capable of producing IL-17 in RA patients and healthy controls.

## Materials and Methods

### Patients and Controls

Peripheral blood samples were collected from 20 RA patients full-filling the 1991 revised criteria for the classification of RA of the American College of Rheumatology (ACR) [34], as well as from 20 healthy volunteer controls. RA patients consisted of 5 men and 15 women with a mean age of 56.7 years (range, 26-79 years) and a median duration of the disease of 10.2 years (range, 6 months- 36 years) at the time of blood drawing. The median disease activity score (DAS28) was 2.89 (range 1.0-4.1). 16 RA patients were treated with corticosteroids, 7 with methotrexate and 8 received other immunosuppressive therapies at the time of blood drawing. The group of healthy donors consisted of 12 men and 8 women, whose median age was 29.2 years (range 20-47 years). All controls were healthy at the time of blood donation. The study was approved by the Ethics Committee of the Medical Faculty, University of Tübingen according to the Declaration of Helsinki principals (13/2005V). All patients and controls provided written informed consent.

### B cell lines

The EBV-transformed B cell lines Olga (IHW9071), WT51 (IHW9021), AMAI (IHW9010), Boleth (IHW9031), LD2B (IHW9083) obtained from the 10th International Histocompatibility Workshop (IHW) were expanded in RPMI-1640 supplemented with 2 mM L-glutamine, 100 μg/ml penicillin, 100 μg/ml streptomycin (Roche Diagnostics, Mannheim, Germany), and 10% heat inactivated FCS (Sigma Aldrich, Taufkirchen, Germany).

### Mononuclear Cell Preparations

Peripheral blood mononuclear cells (PBMC) were isolated from blood samples (heparinized blood) by Ficoll-Hypaque (Amersham, Freiburg, Germany) density gradient centrifugation. Isolated cells were cryopreserved in RPMI-1640 medium (Invitrogen Life Technologies, Karlsruhe, Germany) with 10% dimethylsulfoxide, 20% fetal calf serum (FCS; Sigma Aldrich, Taufkirchen, Germany) and stored in liquid nitrogen until analysis.

10^6^ isolated thawed PBMC were incubated either with 12.5 ng phorbol-12-myristate-13-acetate (PMA) (Gibco, Karlsruhe, Germany) and 0.5 µg ionomycin (i) (Sigma Aldrich, Taufkirchen, Germany) or with 5 µg phytohemagglutinin (PHA) (Invitrogen Life Technologies, Karlsruhe, Germany) or with a pool of 23 peptides of MHC-class II-restricted T cell epitopes of influenza A and B virus, cytomegalovirus, Epstein-Barr virus and tetanus toxin at a concentration of 2 µg/peptide (Panatecs, Tübingen, Germany) per 10^6^ cells for 20 h at 37° C and 5% CO_2_ in RPMI-1640 medium. Unstimulated cells were kept in RPMI-1640 medium at 5% CO_2_ and 37° C also for 20 h as negative controls. After 4 h of stimulation, cytokine secretion was blocked by the addition of 10 µg brefeldin A (Sigma Aldrich, Taufkirchen, Germany) to the cell cultures.

### Multi-Parameter Flow Cytometry

Multi-colour flow cytometry, cell surface immunophenotyping of PBMC and of B cell lines was systematically performed in FACS buffer (PBS, 0.1% BSA, 0.0025% NaN_3_) with the following optimally pre-titrated fluorochrome-conjugated monoclonal antibodies: anti-CD3-FITC, anti-CD8-PerCP, anti-CD56-Alexa700, anti-IFN-γ-PE-Cy7 (BD, Heidelberg, Germany), anti-CD4-Pacific- Blue, anti-CD25-APC-Cy7, anti-CD28-APC, anti-CD56-Alexa700, anti-CD19-PerCP, anti-CD11b-PE, anti-CD14-PE-Cy7, anti-CD19-BV421, anti-CD20-BV570,anti-CD24-PE-Cy7, anti-CD27-BV605, anti-CD38-BV711, anti-IgD-FITC (BioLegend, Biozol, Eching, Germany), anti-CD3-Q.655 (Invitrogen Life Technologies, Karlsruhe, Germany) and anti-CD20-FITC (Immunotools, Friesoythe, Germany). In each sample, dead cells were discriminated from live cells by the addition of ethidium monoazide (EMA, Invitrogen, Karlsruhe, Germany). Before specific surface labelling, human immunoglobulin Gamunex (Bayer, Leverkusen, Germany) was added to each sample for Fc-receptor blockade. Subsequently, cells were washed, fixed and permeabilized using Cytofix/Cytoperm reagent (BD, Heidelberg, Germany) for intracellular analysis of IL-17 applying anti-IL-17-PE (Invitrogen, Karlsruhe, Germany) or anti-IL-17-PerCP-Cy5.5 (eBioscience, Frankfurt, Germany). All events (at least 10^5^ cell events) were acquired directly after staining on a BD LSRII (BD Biosciences, Heidelberg, Germany) flow-cytometer using FACSDiva software (BD Biosciences).

For each experiment, CompBeads anti-mouse Ig κ-chain (BD Biosciences) were stained with the applied fluorescence-labelled murine monoclonal antibodies as positive controls and BD Comp Beads Negative Control (BD Biosciences) were used as negative control reagents for compensation. Unstained cells, treated under the same experimental conditions, also served as negative controls. In a subset of patients and controls, matched isotype controls (mouse IgG1 or IgG2b), IL-17 single stained controls and FMO controls were included. 

The compensation corrections of spectral overlaps of the fluorochrome-labelled antibody sets applied was calculated by Flow Jo 7.2.2 (TreeStar Inc, San Carlos, CA, USA). 

**Figure 1:**
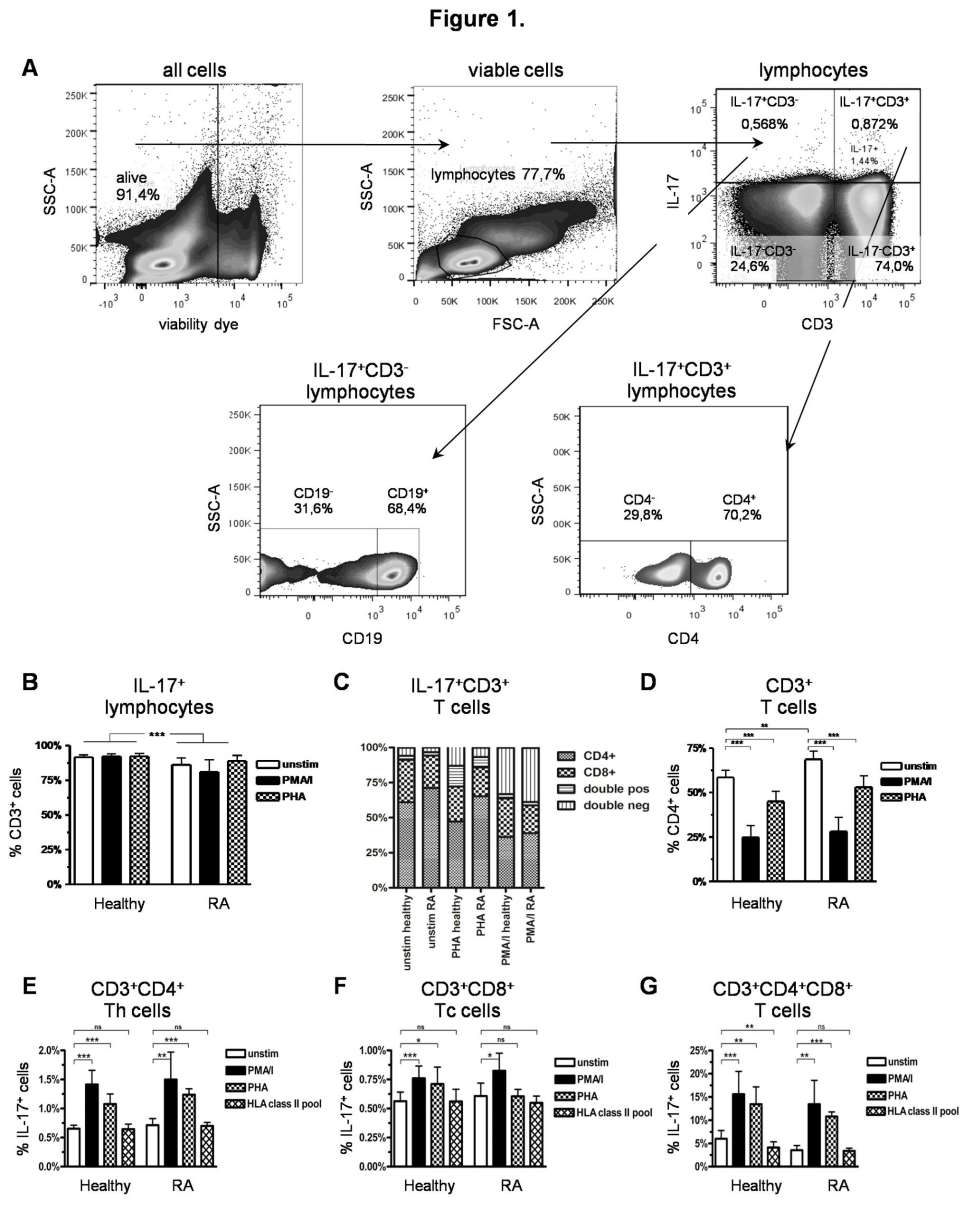
Gating strategy and IL-17^+^ T cells in RA patients and healthy controls. A) Exemplary gating strategy shown on representative pseudo-colour dot-plots of unstimulated PBMC of a RA patient. Dead cells were excluded using EMA-viability dye. Lymphocytes were gated (FSC vs. SSC), followed by the subgating of CD3^+^ and IL-17^+^ cells. IL-17^+^CD3^+^ T cells and IL-17^+^CD3^-^ non-T cells were further specified. Frequencies in each subgate are expressed as percentage of their parent population; B-D) Comparative analysis of CD3^+^ lymphocytes in PBMC stimulated with PMA/i, PHA or incubated in RPMI-1640 without stimulation for 20 h in RA patients and healthy controls; B) Percentage of CD3^+^ T cells within the IL-17^+^ lymphocytes; C) Percentage of CD4^+^ and CD8^+^ cells within the IL-17^+^CD3^+^ T cell population, D) Percentage of CD4^+^ cells within CD3^+^ T cells; E-F) Comparative analysis of IL-17 expression in different T cell populations stimulated with PMA/i, PHA, a pool of 23 peptides of MHC-class II-restricted T cell epitopes or incubated in RPMI-1640 without stimulation for 20 h; E) Percentage of IL-17^+^ cells within CD3^+^CD4^+^ Th cells; F) Percentage of IL-17^+^ cells within CD3^+^CD8^+^ Tc cells; G) Percentage of IL-17^+^ cells within CD3^+^CD4^+^CD8^+^ double positive T cells. Data from healthy controls (n = 20) and RA patients (n = 20). *p < 0.05; **p < 0.01, ***p < 0.001,ns = not significant, column represents the mean and bars indicate 95% CI.

For data analysis using the Flow Jo 7.2.2 software, the populations were gated in sequence. EMA-negative cells were selected in FSC-A vs EMA dot-plots to exclude dead cells as shown in Figure 1A. Subsequently, lymphocytes were gated based on characteristic properties of the cells in forward and side scatter. T cell subpopulations were then selected in the CD3/SSC plot by their bright positivity as the CD3^+^ subset or by their lack of reactivity as the CD3^-^ subset, and evaluated for the expression of IL-17 and CD4. Alternatively, IL-17^+^ cells were first gated in the IL-17/CD3 plots and analyzed as IL-17^+^CD3^+^ T cells or IL-17^+^CD3^-^ non-T cells for CD4^+^, CD8^+^, CD56^+^ or CD19^+^ lymphocyte subsets as well as CD11b^+^ or CD14^+^ monocytes. The B cell gating was performed as previously described [35]. Different fluorochrome-stained populations were highlighted by XY-quadrants and gates to obtain the corresponding statistics including counted events, percentage of parent, percentage of total and means.

### Immunomagnetic Cell Sorting of CD19^+^ B cells and CD4^+^ Th cells

Th cells and B cells were positively isolated from fresh PBMC of healthy donors and freshly thawed PBMC of RA patients using CD4 and CD19 MicroBeads (Miltenyi, Bergisch Gladbach, Germany) with a MiniMacs Separator in combination with MS columns according to the manufacturer's instructions. The purity of the cells after separation was >87% as determined by FACS analysis. 

### Polymerase Chain Reaction

RNA isolation was performed using RNeasy mini kits (Qiagen, Hilden, Germany). cDNA synthesis was done from 500 µg RNA of each sorted cell population using SuperScript III (Invitrogen, Karlsruhe, Germany) according to the manufacturer's instructions. A nested PCR was applied to determine IL-17 mRNA expression with the following two primer sets: set I: forward, 5'-ATG ACT CCT GGG AAG ACC TCA TTG-3`, reverse, 5'-TTA GGC CAC ATG GTG GAC AAT CGG-3'; nested set II: forward, 5'-AAT CTC CAC CGC AAT GAG GA-3', reverse, 5'-CGT TCC CAT CAG CGT TGA TG-3'. IL-17A was amplified in 37 cycles (56°C) with the primer set I followed by a nested PCR with the primer set II in 32 cycles. The Puc mix marker 8 (Fermentas, St. Leon-Rot, Germany) was used. The expected 94 bp amplification product was detected by agarose gel-electrophoresis and verified by sequencing.

### Statistical Analysis

Statistical analysis and graphical display of the results were performed using GraphPad Prism v4.03 (GraphPad Software, San Diego California USA). Mann-Whitney U-tests and Wilcoxon signed rank tests were used to compare two unpaired or paired data sets where appropriate. A p value of less than 0.05 was considered to be statistically significant. Spearman's rank correlation was used to quantify the extent of statistical dependence between IL-17 and clinical parameters.

## Results

### Heterogeneity of IL-17-producing T cells in RA patients and healthy controls

To evaluate the frequency and cellular source of IL-17 in RA, IL-17^+^CD3^+^ T cells were compared upon reactivity to different stimuli in RA patients and healthy controls. A representative example of the flow-cytometric gating strategy in viable PBMC of a RA patient is shown in Figure 1A. Most of the IL-17^+^ lymphocytes were present within the CD3^+^ T cell subset in both stimulated and unstimulated PBMC of RA patients and healthy controls (Figure 1B). The RA patients constitutively had a significantly higher proportion of IL-17^+^CD3^+^CD4^+^ Th17 cells than the healthy controls (Figure 1C), but also showed an overall significantly increased CD4^+^ T-helper cell fraction of the CD3^+^ T cells (Figure 1D). The higher percentages of IL-17^+^CD3^+^CD4^+^ Th17 cells thus appeared to reflect a general increase of the CD3^+^CD4^+^ T-helper cells in RA patients. On the contrary, the fraction of IL-17-expressing CD3^+^CD4^+^ T-helper cells tended to be only slightly higher in RA patients than in the healthy controls (Figure 1E). These results are consistent with prevoius reports of other groups [27,29].

IL-17^+^CD3^+^ T cells were also evaluated after stimulation with PMA/ionomycin (PMA/i), PHA or a pool of peptides of MHC class II-restricted T cell epitopes. In RA patients and healthy controls, the proportion of CD4^+^ cells within IL-17^+^CD3^+^ T cells (Figure 1C) or CD4^+^ T-helper cells within CD3^+^ T-lymphocytes (Figure 1D) decreased significantly after PMA/i stimulation due to down-regulation of CD4 [36]. This was also observed after PHA activation. The fraction of IL-17^+^ cells was nevertheless increased significantly among the CD3^+^CD4^+^ helper T cells after mitogen stimulation (Figure 1E). Stimulation with PMA/i led to an even higher IL-17^+^ cell fraction than PHA with no significant difference between patients and controls.

Other IL-17-producing T cells were also identified, thus, small numbers of CD3^+^CD8^+^ IL-17^+^ Tc cells (Figure 1C + F) were present at similar frequencies in patients and controls. Presence of an IL-17^+^ CD8^+^ T cell subset had previously been detected in lung tissue of patients with chronic obstructive pulmonary disease [37]. In the present study, also a small fraction of CD3^+^CD4^+^CD8^+^ double-positive T cells was seen after stimulation. These represented a notable proportion (2.3%-14.9%) of the total IL-17-expressing CD3^+^ T cell population especially after PMA/i or PHA stimulation (Figure 1C). Double-negative CD4^-^CD8^-^ IL-17^+^CD3^+^ T cells which were also seen in controls and patients (Figure 1C) probably included γδ T cells or CD4^-^CD8^-^ αβ T cells expressing IL-17, as previously described [12,14,38], although CD4 down-regulation after stimulation with PHA or PMA/I cannot be entirely excluded.

Chronic infection has been repeatedly suggested to play an important role in the development of RA [39]. We used a pool of MHC II-restricted peptides from viral proteins of EBV, CMV, influenza A, influenza B and tetanus toxin to stimulate IL-17 production in PBMC. These experiments did not reveal major changes of the IL-17^+^CD3^+^ T cell frequencies in either RA patients or controls (Figure 1E-G). The only change was a significant decrease of IL-17^+^ double-positive CD3^+^CD4^+^CD8^+^ T cells observed in healthy individuals after incubation with the peptide pool (Figure 1G).

### IL-17 producing non-T cells

**Figure 2:**
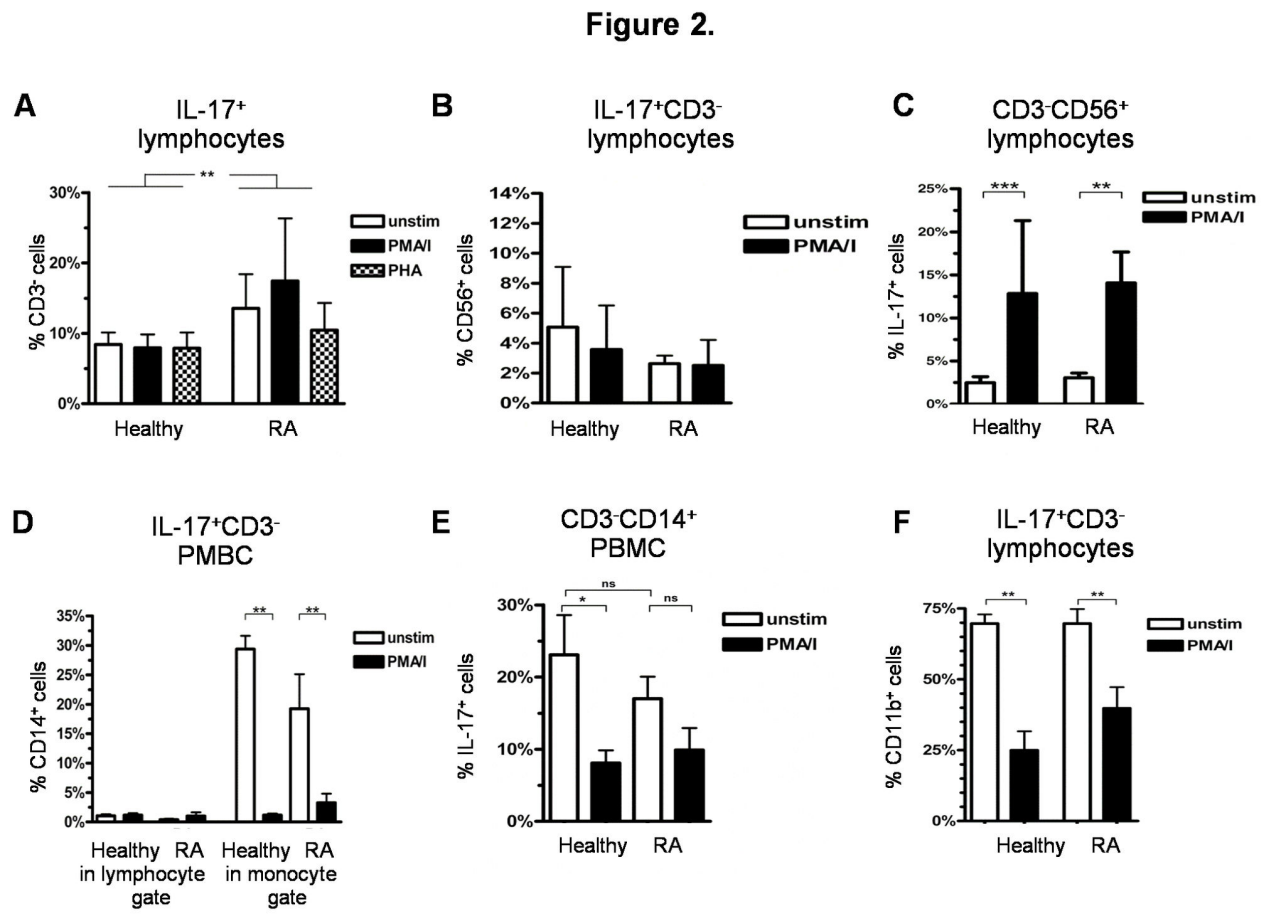
IL-17^+^ non-T cells. A-F) Comparative analysis of PBMC stimulated with PMA/i for 20h or incubated in RPMI-1640 medium with the addition of Brefeldin A for 16 h in RA patients and healthy controls; A) Percentage of CD3^-^ non-T cells within IL-17^+^ lymphocytes (RA n = 20, healthy controls n = 20); B) Percentage of CD56^+^ NK cells within IL-17^+^CD3^-^ non-T cells; C) Percentage of IL-17^+^ cells within CD3^-^CD56^+^ NK cells; D) Percentage of CD14^+^ monocytes in IL-17^+^CD3^-^ non-T cells within the lymphocyte gate and within the monocyte gate (FSC vs. SSC); E) Percentage of IL-17^+^ cells within CD3^-^CD14^+^ monocytes within the monocyte gate; F) Percentage of CD11b^+^ cells within IL-17^+^CD3^-^ lymphocytes. Data B)-F) PMBC of healthy controls (n = 8) and RA patients (n = 8). *p < 0.05, **p < 0.01, ***p < 0.001, ns = not significant, column represents the mean and bars indicate 95% CI.

PBMC of RA patients revealed a significantly larger IL-17^+^CD3^-^ non-T cell fraction compared to healthy controls (Figure 2A, p < 0.01). The nature of these IL-17-producing non-T cells was next investigated. First, the previously described IL-17-producing CD3^-^CD56^+^ NK cells [3], were found to represent a small proportion of the IL-17^+^CD3^-^ non-T cell populations (Figure 2B). There was a tendency towards higher proportions of these cells in healthy controls compared to RA patients. Within these CD3^-^CD56^+^ NK cells, IL-17^+^ lymphocytes increased significantly on stimulation with PMA/i (Figure 2C), but to the same extent in both RA patients and healthy controls. A small proportion of the IL-17^+^ non-T cells also appeared to be CD3^-^CD14^+^ monocytes remaining within the lymphocyte gate used for standardized evaluation in these analyses (Figure 2D). Presence of an IL-17^+^ monocyte subset in PBMC could be confirmed by specifically gating on monocytes in SSC vs. FSC. Healthy controls were found to possess an even larger proportion of these IL-17^+^CD3^-^CD14^+^ monocytes than RA patients (Figure 2E). In contrast to NK cells, PMA/i stimulation led to a significant decrease of IL-17^+^ cells in the monocyte gate (Figure 2E), but also decreased the CD14^+^ cell fraction of the IL-17^+^CD3^-^ population significantly (Figure 2D). 

Interestingly, up to 70% of all unstimulated IL-17^+^CD3^-^ non-T cells expressed CD11b, the alpha chain of the heterodimeric integrin alpha-M beta-2 (αMβ2) expressed on monocytes, macrophages, granulocytes, follicular dendritic cells, natural killer cells, and even some T/B cells. Upon PMA/i stimulation, this cell fraction also decreased significantly in healthy persons and in RA patients (Figure 2F). Among these IL-17^+^CD3^-^CD11b^+^ cells, CD14^+^ monocytes, as well as CD19^+^ B cells, were identified (data not shown). In addition to B cells and monocytes, a respectable number of IL-17^+^CD3^-^CD11b^+^ non-T cells could not be further characterized with the antibody sets used here.

### IL-17 expressing B cells

**Figure 3:**
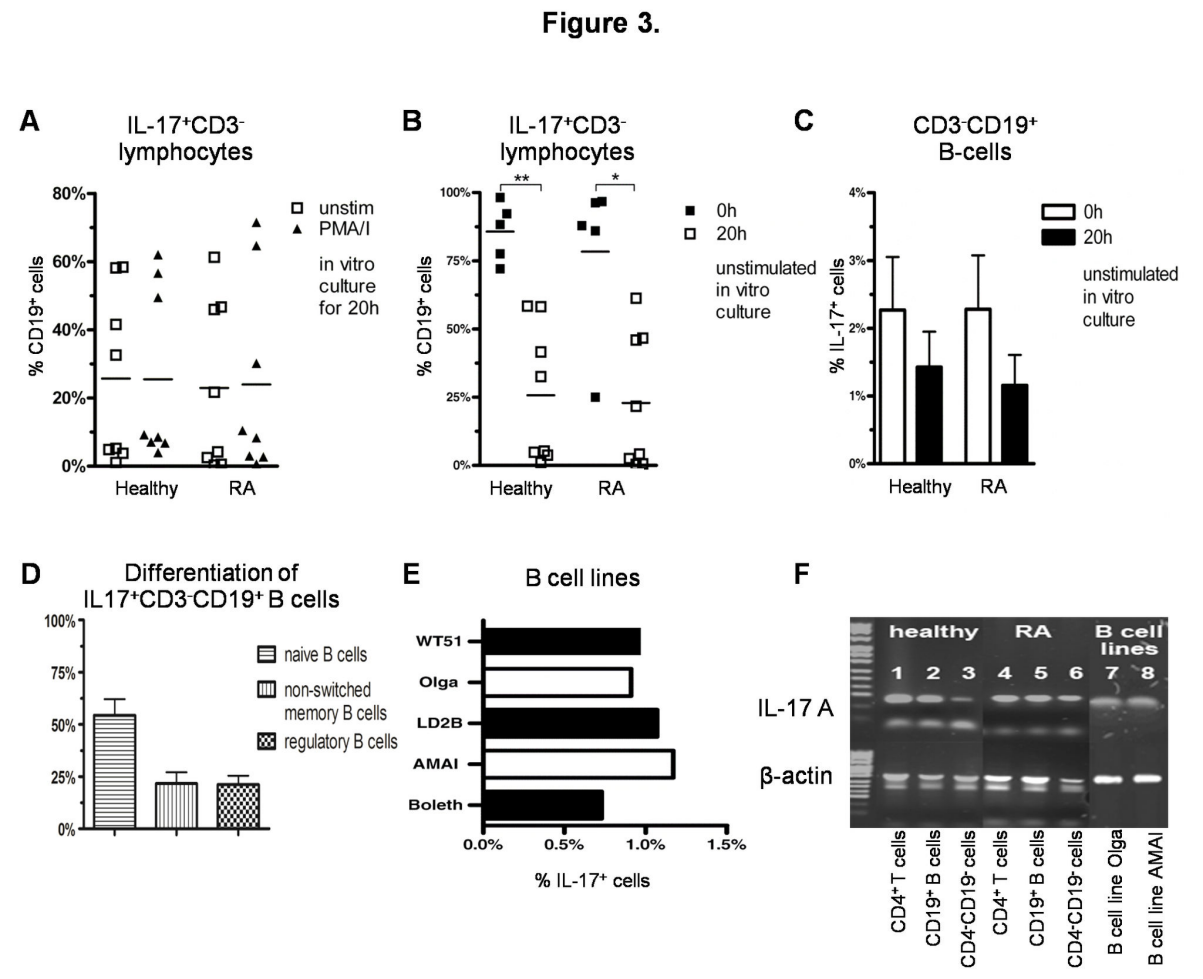
IL-17 expressing B cells. A) Percentage of CD19^+^ B cells within IL-17^+^CD3^-^ lymphocytes in RA patients (n = 8) and healthy controls (n = 8) in unstimulated PBMC or after stimulation with PMA/i for 20 h; B+C) freshly thawed PBMC or PBMC cultured for 20 h in RPMI-1640 medium with the addition of Brefeldin A for 16h (n = 5 and n = 8, respectively) in RA patients and healthy controls; B) Percentage of CD19^+^ B cells within IL-17^+^CD3^-^ lymphocytes; C) Percentage of IL-17^+^ cells within CD3^-^CD19^+^ B cells; D) Percentage of IgD^+^CD27^-^ naïve B cells, IgD^+^CD27^+^ non-switched memory B cells and CD24^++^CD38^++^ regulatory B cells of IL-17^+^CD3^-^CD19^+^ B cells of freshly thawed PBMC of healthy controls (n=3); E) Percentage of IL-17^+^ cells in EBV-transformed B cell lines without stimulation; F) Representative gel-electrophoresis of amplificates of IL-17A RT-PCR of unstimulated freshly thawed CD4^+^ and CD19^+^ MACS-sorted cells of a healthy donor: CD4^+^ T cells (lane 1), CD19^+^ B cells (lane 2), CD4^-^CD19^-^ cells (lane 3); of a RA patient: CD4^+^ T cells (lane 4), CD19^+^ B cells (lane 5), CD4^-^CD19^-^ cells (lane 6); and of the EBV-transformed B cell lines: Olga (lane 7), AMAI (lane 8); upper level: amplificates of IL-17A, lower level: corresponding control amplificates of β-actin, Marker: Puc 8 mix ladder; The data shown are representative results of RT-PCR of 3 healthy donors and 3 RA patients. *p < 0.05; **p < 0.01, ns = not significant; columns represent the mean and bars indicate 95% CI.

A large population of IL-17^+^CD3^-^ non-T cells was surprisingly found to consist of CD19^+^ B cells both in RA patients and healthy controls. To our knowledge this is the first description of IL-17 producing B cells in PBMC. The B cell subset could account for up to a mean of 25% of the IL-17^+^CD3^-^ non-T cells (Figure 3A), although with highly variable percentages in healthy individuals (range 0.6% - 62%, n = 8) as well as in RA patients (range 0.3% - 71.5%, n = 8) independent of their disease state and treatment after short term in vitro culture. The variability of this subset was much smaller when examined directly ex vivo, and most of the IL-17^+^CD3^-^ non-T cells were found to express CD19 (Figure 3B). Moreover, after stimulation with PMA/i, the B cell fraction of the IL-17^+^CD3^-^ non-T cells did not change (Figure 3A). The IL-17^+^ fraction of B-cells, however, decreased considerably after ex vivo culture (Figure 3C). We further stained 3 healthy donors with B cell specific antibodies and IL-17 to further differentiate those IL-17 producing B cells. 96% of IL-17^+^CD3^-^CD19^+^ B cells expressed CD20^+^ (data not shown). More than half of the of IL-17^+^CD3^-^CD19^+^ B cells were CD27^-^IgD^+^ naïve B cells while the rest accounted for up to 21% of CD27^-^IgD^+^ non-switched memory B cells and 21% of CD24^++^CD38^++^ regulatory B cells (Figure 3D). IL-17 expression was similarly investigated in five established EBV-transformed B cell lines. A subset of IL-17^+^ cells (0.73% - 1.17%) was also observed in all analyzed B cell lines (Figure 3E), which concomitantly expressed CD20 and CD11b (data not shown).

To further confirm IL-17 mRNA expression in B cells subsets, IL-17 RT-PCR was performed on EBV-transformed B cell lines as well as on CD4 and CD19-specific immunomagnetic bead-enriched lymphocytes from PBMC of healthy donors and RA patients (Figure 3F). The highest amount of IL-17 transcripts was detected within the CD4^+^ sorted Th cells, followed by the CD19^+^ sorted B cells with no major differences between RA patients and healthy donors. Only a small amount of IL-17 mRNA was detected in those negatively enriched remaining peripheral blood cells. The presence of IL-17 mRNA in B cell lines was also confirmed by RT-PCR for the tested EBV-transformed B cell lines Olga and AMAI (Figure 3F). The IL-17 specificity of the detected amplification products was verified by sequencing.

### Influence of in vitro culture on the differentiation of IL-17^+^ cells

**Figure 4:**
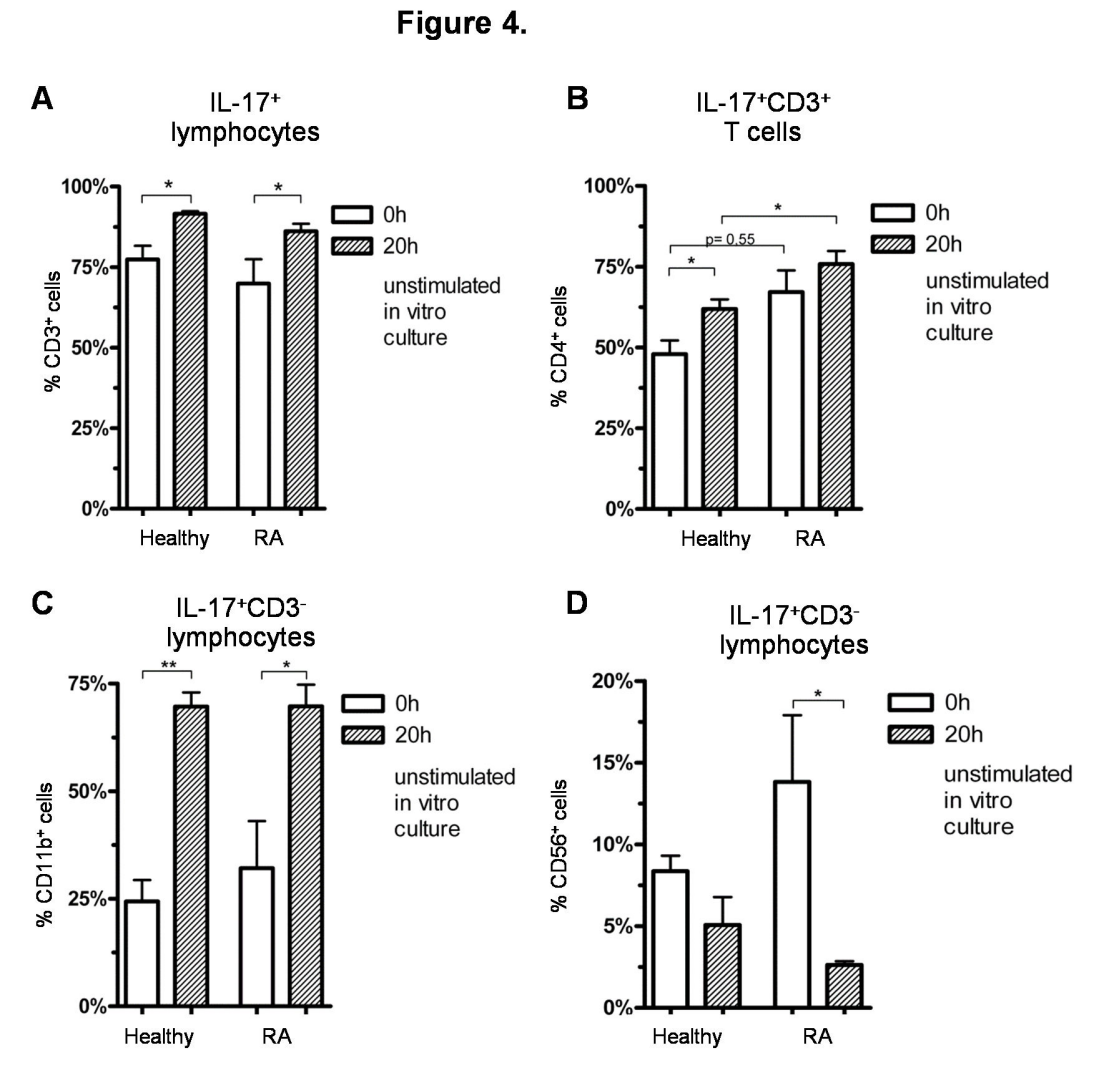
Influence of in vitro culture on IL-17^+^ cells. A-D) Comparison of freshly thawed cells and cells cultured for 20 h in RPMI-1640 medium with the addition of Brefeldin A for 16 h (n = 5 and n = 8, respectively) in RA patients and healthy controls; A) Percentage of CD3^+^ T cells within IL-17^+^ lymphocytes; B) Percentage of CD4^+^ cells within IL-17^+^CD3^+^ T cells; C) Percentage of CD11b^+^ cells within IL-17^+^CD3^-^ lymphocytes; D) Percentage of CD56^+^ NK cells within IL-17^+^CD3^-^ lymphocytes. *p < 0.05, **p < 0.01,***p < 0.001, ns = not significant, columns represent the mean and bars indicate 95% CI.

Because in vitro culture could influence detection of IL-17^+^ cells, we compared the different IL-17-expressing cell populations described above in PBMC ex vivo to those cultured for 20 h in RPMI-1640 medium with the addition of brefeldin A for 16h. After in vitro culture, a significant increase of the percentages of IL-17^+^CD3^+^ T cells (Figure 4A), IL-17^+^CD3^+^CD4^+^ Th cells (Figure 4B) and IL-17^+^CD3^-^CD11b^+^ cells (Figure 4C) was observed, which appeared to correspond to a significant decrease of the IL-17^+^CD3^-^CD19^+^ B cells (Figure 3B) and IL-17^+^CD3^-^CD56^+^ NK cells (Figure 4D) subsets. No influence of in vitro culture was seen on the frequencies of the IL-17^+^CD3^-^CD14^+^ monocytes (data not shown). 

## Discussion

Rheumatoid arthritis is a chronic inflammatory autoimmune disease in the pathogenesis of which IL-17 is thought to play a key role. Previous analyses of Th17 in human inflamed synovial tissues and blood as well as measurements of IL-17 levels in synovial fluid [24,27,40] and seruum [29,33,41], have provided disparate results which still do not allow unequivocal conclusions on the cellular source and pathogenetic role of IL-17 in human RA. The present study shows that in RA patients IL-17 is also derived from a substantial number of cells other than the Th17 subset which include NK cells, other innate immune cells and in particular B lymphocytes in peripheral blood. The present analysis also provides data that differences in the detection of IL-17^+^ cells in former studies might be due to differences in the applied in vitro cultures.

In accordance with previous analyses [27,42], we found only slightly raised frequencies of IL-17^+^ lymphocytes in PBMC of RA patients relative to healthy controls. Furthermore, neither the observed IL-17^+^CD3^+^ T cell populations in general nor the included CD4^+^ Th17 cells although slightly elevated in RA were significantly different in patients and controls as had been previously reported [27]. The Th17 cell fractions were most likely concomitantly augmented as a result of the observed elevation of the whole subset of CD3^+^CD4^+^ T lymphocytes in RA patients. The IL-17^+^ cells also could be significantly expanded with mitogens such as PMA/i or PHA, but not with a viral peptide pool, in both RA patients and healthy individuals, as might be expected. These findings suggest no major abnormalities in the differentiation of the Th17 cells in the RA patients.

Most patients included in this study suffered from disease of long duration with well-controlled low or moderate activity as reflected by the level of C-reactive protein, leukocyte count, and DAS28 score. This could account for the minor differences seen in the circulating IL-17 populations between patients and controls and may explain results of other authors at variance with our study. Analysis of treatment-naïve patients with early RA had shown significantly higher percentages of peripheral Th17 cells compared to healthy controls [31]. In addition, most of the patients in the present study were also treated with corticosteroids and/or methotrexate, which are known to decrease IL-17 levels [43-45]. Moreover, peripheral Th17 levels also failed to correlate with other clinical parameters of disease activity and severity (data not shown) as had been observed previously [27,29,42]. These findings indicate that IL-17^+^CD3^+^ T lymphocyte levels including Th17 cells in peripheral blood in particular may not be informative of the disease status later during RA.

In the present study we also focused on in vitro alterations of IL-17 producing cells. After short-term culture in which the lymphocyte responses to different stimuli were tested, a significantly higher proportion of CD3^+^ T cells and CD3^+^CD4^+^ Th17 cells within the IL-17^+^ lymphocyte population, but a relative decline of IL-17^+^CD3^-^ non-T cells was observed in unstimulated control samples of PBMC, in comparison to direct ex vivo analysis. Thus, in our study in vitro culture might have even led to an overestimation of the amount of CD3^+^ T cells and CD3^+^CD4^+^ Th17 subsets expressing IL-17, but underestimation of in vivo frequencies of IL-17^+^CD3^-^ non-T lymphocytes, such as the B cells, monocytes and NK cells. Similar culture systems have been used frequently in previous analyses of IL-17^+^ cells in RA and could also have influenced the predominant and/or variable detection of T cells as major IL-17-producing cells in diseased and healthy individuals in previous studies.

In contrast to the Th17 cells, we observed that IL-17 expressing CD3^-^ non-T cells were significantly elevated in PBMC of RA patients compared to healthy controls. This applied to the directly ex vivo tested PBMC as well as to the short-term cultured samples. Recent reports of local effector cells in joints of established RA have provided first evidence that innate immune cells, particularly mast cells may serve as another major source of IL-17A in RA and contribute to pro-inflammatory effector responses in the arthritic synovium [30,40]. IL-17 producing T cells, however, were variably and often rarely observed in the inflamed human synovium [29,30,40]. These unexpected findings were related to the plasticity of Th17 cells and their phenotypic changes upon tissue entry and/or over the course of the disease. In the present study, up to 30% of IL-17^+^ PBMC represented IL-17^+^CD3^-^ non-T cell subpopulations in the peripheral blood of RA patients, significantly different to healthy individuals. Among these, NK cells, monocytes and, rather surprisingly, B cells were identified. Therefore, these data also suggest that diff**e**rent adaptive and innate immune cells can all contribute to IL-17A production in RA. Our observations thus further support the view that additional innate immune cells are important effectors of IL-17- driven chronic inflammatory pathways in RA. However, it still needs to be determined whether, such IL-17^+^CD3^-^ non-T cells preferentially localize to synovial tissue and participate in the pathology of the inflamed joints at various stages of the disease. To the best of our knowledge, in this study we show for the first time that subsets of CD19^+^ B cells express IL-17A in PBMC of RA patients and healthy individuals. Although the signalling pathways and interactions of B cells in RA are still incompletely understood, they are known to have a pivotal role in the pathogenesis of RA in humans and animal models [46]. It has been shown that autoantigen-specific B cells can act as antigen-presenting cells equally as efficiently as dendritic cells, and thus drive activation of auto-reactive CD4^+^ T cells in RA [47-50]. In this study, about 96% of the IL17^+^ producing CD3^-^CD19^+^ B cells coexpressed CD20, a specific surface molecule of mature naïve and antigen-reactive, but not plasma B cells, which is effectively used as a target structure for therapeutic modulation and/or depletion of B cells by monoclonal antibodies such as Rituximab. Most of the IL-17^+^ B cells (54%) in the healthy controls of this study seemed to represent CD19^+^CD27^-^IgD^+^ naïve B cells. Naïve B cells were shown to produce proinflammatory cytokines in response to B cell receptor and CD40 stimulation [51]. Another large fraction of the IL-17+ B cells (21%) were CD19^+^CD24^++^CD38^++^ regulatory B cells which have been shown to differentiate into naïve B cells, switched and non-switched memory B cells upon CpG stimulation via TLR9 [52]. Regulatory B cells are capable to suppress Th1 and Th17 differentation [53]. Flores-Borjaet et al. showed that in RA patients regulatory B cells failed to suppress differentiation of Th17 cells [54]. Furthermore, CD19^+^IgD^+^CD27^+^ non-switched memory B cells also accounted for up to 21% of the IL-17^+^ B cells of this study. Non-switched B cells have been reported to accumulate in the synovium of RA patients and to respond to TNF-α treatment [55]. 

Unfortunately, we could not clarify stimulation of IL-17 in B cells in this study. PMA/i failed to stimulate IL-17^+^CD3^-^CD19^+^ B cells in vitro, but led to an increase of IL-17 in CD3^+^CD4^+^ Th17 cells. So far the bacterium T. cruzi today is the only known trigger of IL-17 production in B cells [19]. It was shown to depend on a parasite trans-sialidase activity which modifies the cell-surface CD45 and thus initiates a new signalling program independent of RORγt, RORα or AhR for the stimulation of IL-17. Neither IL-6, IL-23 nor MyD88-associated TLR signalling were relevant for this IL-17 production in B cells [56]. The B cell activating factor activating factor (BAFF), a member of the tumor necrosis superfamily, has been recognized to positively influence proinflammatory cytokine production in B cells and might also be capable to stimulate IL-17 production in B cells. BAFF seems to play a pivotal role in different autoimmune diseases including RA [57]. In RA, increased BAFF levels have been observed to correlate with high levels of the rheumatoid factor [58], with clinical disease activity, and response to treatment in early rheumatoid arthritis [59]. In a clinical phase II trial the blockade of BAFF with a neutralising antibody called Tabalumab provided promising results in patients with active rheumatoid arthritis (RA) [60]. BAFF gene silencing has been shown to lead to a significant reduction of IL-17 and Th17 cells [61]. In addition, had Doreau et al. observed that IL-17 alone, but together with BAFF even with higher efficiency, could promote proliferation and differentiation of B-cells and prolong their survival [62]. These findings suggest complex mutual interactions of BAFF and IL-17 on the regulation of B cell differentiation and function. 

Further studies are needed to elucidate signalling pathways for the expression of IL-17A in B cells.

Our data suggest that B cells could also participate in dysregulated IL-17A production and enhance chronic inflammatory innate immune responses as well as erosive arthritis in RA. Thus, clinical effects of today´s B cell-directed therapies in RA could be also mediated by influences on IL-17 production and levels in peripheral blood and tissues. Known beneficial clinical responses of B cell depletion with Rituximab (anti-CD20-antibody) in several clinical trials have been reviewed by Korhonen et al. [63] recently.

In conclusion, our study provides evidence that in addition to Th17 cells, a substantial number of other innate and adaptive immune cells, in particular also B cells participate in the IL-17production during established RA and may thus influence disease activity and joint destruction.

## References

[1] Rouvier E, Luciani MF, Mattéi MG, Denizot F, Golstein P (1993) CTLA-8, cloned from an activated T cell, bearing AU-rich messenger RNA instability sequences, and homologous to a herpesvirus Saimiri gene. J Immunol 150: 5445-5456. PubMed: 8390535. 8390535

[2] Wright JF, Bennett F, Li B, Brooks J, Luxenberg DP et al. (2008) The human IL-17F/IL-17A heterodimeric cytokine signals through the IL-17RA/IL-17RC receptor complex. J Immunol 181: 2799-2805. PubMed: 18684971. 18684971 10.4049/jimmunol.181.4.2799

[3] Weaver CT, Hatton RD, Mangan PR, Harrington LE (2007) IL-17 Family Cytokines and the Expanding Diversity of Effector T Cell Lineages. Annu Rev Immunol 25: 821-852. doi:10.1146/annurev.immunol.25.022106.141557. PubMed: 17201677. 17201677

[4] Umemura M, Yahagi A, Hamada S, Begum MD, Watanabe H et al. (2007) IL-17-mediated regulation of innate and acquired immune response against pulmonary Mycobacterium bovis bacille Calmette-Guerin infection. J Immunol 178: 3786-3796. PubMed: 17339477. 17339477 10.4049/jimmunol.178.6.3786

[5] Harrington LE, Hatton RD, Mangan PR, Turner H, Murphy TL et al. (2005) Interleukin 17-producing CD4+ effector T cells develop via a lineage distinct from the T helper type 1 and 2 lineages. Nat Immunol 6: 1123-1132. doi:10.1038/ni1254. PubMed: 16200070. 16200070

[6] Park H, Li Z, Yang XO, Chang SH, Nurieva R et al. (2005) A distinct lineage of CD4 T cells regulates tissue inflammation by producing interleukin 17. Nat Immunol 6: 1133-1141. doi:10.1038/ni1261. PubMed: 16200068. 16200068 PMC1618871

[7] Ivanov II, McKenzie BS, Zhou L, Tadokoro CE, Lepelley A et al. (2006) The orphan nuclear receptor RORgammat directs the differentiation program of proinflammatory IL-17+ T helper cells. Cell 126: 1121-1133. doi:10.1016/j.cell.2006.07.035. PubMed: 16990136. 16990136

[8] Veldhoen M, Hocking RJ, Atkins CJ, Locksley RM, Stockinger B (2006) TGFbeta in the context of an inflammatory cytokine milieu supports de novo differentiation of IL-17-producing T cells. Immunity 24: 179-189. doi:10.1016/j.immuni.2006.01.001. PubMed: 16473830. 16473830

[9] Mangan PR, Harrington LE, O'Quinn DB, Helms WS, Bullard DC et al. (2006) Transforming growth factor-beta induces development of the T(h)17 lineage. Nature 441: 231-234. doi:10.1038/nature04754. PubMed: 16648837. 16648837

[10] Bettelli E, Carrier Y, Gao W, Korn T, Strom TB et al. (2006) Reciprocal developmental pathways for the generation of pathogenic effector TH17 and regulatory T cells. Nature 441: 235-238. doi:10.1038/nature04753. PubMed: 16648838. 16648838

[11] Kondo T, Takata H, Matsuki F, Takiguchi M (2009) Cutting edge: Phenotypic characterization and differentiation of human CD8+ T cells producing IL-17. J Immunol 182: 1794-1798. doi:10.4049/jimmunol.0801347. PubMed: 19201830. 19201830

[12] O'Brien RL, Roark CL, Born WK (2009) IL-17-producing gammadelta T cells. Eur J Immunol 39: 662-666. doi:10.1002/eji.200839120. PubMed: 19283718. 19283718 PMC2698711

[13] Takatori H, Kanno Y, Watford WT, Tato CM, Weiss G et al. (2009) Lymphoid tissue inducer-like cells are an innate source of IL-17 and IL-22. J Exp Med 206: 35-41. doi:10.1084/jem.20072713. PubMed: 19114665. 19114665 PMC2626689

[14] Bird L (2008) NKT cells: NKT cells join the IL-17 gang. Nature Reviews Immunology 8: 324. doi:10.1038/nri2325.

[15] Michel JJ, Turesson C, Lemster B, Atkins SR, Iclozan C et al. (2007) Identification of an IL-17-producing NK1.1(neg) iNKT cell population involved in airway neutrophilia. J Exp Med 204: 995-1001. doi:10.1084/jem.20061551. PubMed: 17470641. 17470641 PMC2118594

[16] Fujino S, Andoh A, Bamba S, Ogawa A, Hata K et al. (2003) Increased expression of interleukin 17 in inflammatory bowel disease. Gut 52: 65-70. doi:10.1136/gut.52.1.65. PubMed: 12477762. 12477762 PMC1773503

[17] Hoshino A, Nagao T, Nagi-Miura N, Ohno N, Yasuhara M et al. (2008) MPO-ANCA induces IL-17 production by activated neutrophils in vitro via classical complement pathway-dependent manner. J Autoimmun 31: 79-89. doi:10.1016/j.jaut.2008.03.006. PubMed: 18501296. 18501296

[18] Lin AM, Rubin CJ, Khandpur R, Wang JY, Riblett M et al. (2011) Mast cells and neutrophils release IL-17 through extracellular trap formation in psoriasis. J Immunol 187: 490-500. doi:10.4049/jimmunol.1100123. PubMed: 21606249. 21606249 PMC3119764

[19] Bermejo DA, Jackson SW, Gorosito-Serran M, Acosta-Rodriguez EV, Amezcua-Vesely MC et al. (2013) Trypanosoma cruzi trans-sialidase initiates a program independent of the transcription factors RORγt and Ahr that leads to IL-17 production by activated B cells. Nat Immunol 14: 514-522. doi:10.1038/ni.2569. PubMed: 23563688. 23563688 PMC3631452

[20] Lubberts E, Joosten LA, Oppers B, van den Bersselaar L, Coenen-de Roo CJ et al. (2001) IL-1-independent role of IL-17 in synovial inflammation and joint destruction during collagen-induced arthritis. J Immunol 167: 1004-1013. PubMed: 11441109. 11441109 10.4049/jimmunol.167.2.1004

[21] Lubberts E, Koenders MI, van den Berg WB (2005) The role of T-cell interleukin-17 in conducting destructive arthritis: lessons from animal models. Arthritis Res Ther 7: 29-37. doi:10.1186/ar1550. PubMed: 15642151. 15642151 PMC1064899

[22] Chabaud M, Lubberts E, Joosten L, van den Berg W, Miossec P (2001) IL-17 derived from juxta-articular bone and synovium contributes to joint degradation in rheumatoid arthritis. Arthritis Res 3: 168-177. doi:10.1186/ar294. PubMed: 11299057. 11299057 PMC30709

[23] Shahrara S, Huang Q, Mandelin AM 2nd, Pope RM (2008) TH-17 cells in rheumatoid arthritis. Arthritis Res Ther 10: R93. doi:10.1186/ar2477. PubMed: 18710567. 18710567 PMC2575607

[24] Ziolkowska M, Koc A, Luszczykiewicz G, Ksiezopolska-Pietrzak K, Klimczak E et al. (2000) High levels of IL-17 in rheumatoid arthritis patients: IL-15 triggers in vitro IL-17 production via cyclosporin A-sensitive mechanism. J Immunol 164: 2832-2838. PubMed: 10679127. 10679127 10.4049/jimmunol.164.5.2832

[25] Chabaud M, Durand JM, Buchs N, Fossiez F, Page G et al. (1999) Human interleukin-17: A T cell-derived proinflammatory cytokine produced by the rheumatoid synovium. Arthritis Rheum 42: 963-970. doi:10.1002/1529-0131(199905)42:5. PubMed: 10323452. 10323452

[26] Kotake S, Udagawa N, Takahashi N, Matsuzaki K, Itoh K et al. (1999) IL-17 in synovial fluids from patients with rheumatoid arthritis is a potent stimulator of osteoclastogenesis. J Clin Invest 103: 1345-1352. doi:10.1172/JCI5703. PubMed: 10225978. 10225978 PMC408356

[27] Church LD, Filer AD, Hidalgo E, Howlett KA, Thomas AMC et al. (2010) Rheumatoid synovial fluid interleukin-17-producing CD4 T cells have abundant tumor necrosis factor-alpha co-expression, but little interleukin-22 and interleukin-23R expression. Arthritis Res Ther 12: R184. doi:10.1186/ar3152. PubMed: 20929536. 20929536 PMC2991017

[28] Appel H, Maier R, Wu P, Scheer R, Hempfing A et al. (2011) Analysis of IL-17(+) cells in facet joints of patients with spondyloarthritis suggests that the innate immune pathway might be of greater relevance than the Th17-mediated adaptive immune response. Arthritis Res Ther 13: R95. doi:10.1186/ar3370. PubMed: 21689402. 21689402 PMC3218910

[29] Yamada H, Nakashima Y, Okazaki K, Mawatari T, Fukushi JI et al. (2008) Th1 but not Th17 cells predominate in the joints of patients with rheumatoid arthritis. Ann Rheum Dis 67: 1299-1304. PubMed: 18063670. 18063670 10.1136/ard.2007.080341

[30] Hueber AJ, Asquith DL, Miller AM, Reilly J, Kerr S et al. (2010) Mast cells express IL-17A in rheumatoid arthritis synovium. J Immunol 184: 3336-3340. doi:10.4049/jimmunol.0903566. PubMed: 20200272. 20200272

[31] Colin EM, Asmawidjaja PS, van Hamburg JP, Mus AMC, van Driel M et al. (2010) 1,25-dihydroxyvitamin D3 modulates Th17 polarization and interleukin-22 expression by memory T cells from patients with early rheumatoid arthritis. Arthritis Rheum 62: 132-142. doi:10.1002/art.25043. PubMed: 20039421. 20039421

[32] Shen H, Goodall JC, Hill Gaston JS (2009) Frequency and phenotype of peripheral blood Th17 cells in ankylosing spondylitis and rheumatoid arthritis. Arthritis Rheum 60: 1647-1656. doi:10.1002/art.24568. PubMed: 19479869. 19479869

[33] Jandus C, Bioley G, Rivals J-P, Dudler J, Speiser D et al. (2008) Increased numbers of circulating polyfunctional Th17 memory cells in patients with seronegative spondylarthritides. Arthritis Rheum 58: 2307-2317. doi:10.1002/art.23655. PubMed: 18668556. 18668556

[34] Hochberg MC, Chang RW, Dwosh I, Lindsey S, Pincus T, et al. (1992) The American College of Rheumatology 1991 revised criteria for the classification of global functional status in rheumatoid arthritis. Arthritis Rheum 35: 498-502 10.1002/art.1780350502 1575785

[35] Morbach H, Eichhorn EM, Liese JG, Girschick HJ (2010) Reference values for B cell subpopulations from infancy to adulthood. Clin Exp Immunol 162: 271-279. doi:10.1111/j.1365-2249.2010.04206.x. PubMed: 20854328. 20854328 PMC2996594

[36] Weyand CM, Goronzy J, Fathman CG (1987) Modulation of CD4 by antigenic activation. J Immunol 138: 1351-1354. PubMed: 3100638. 3100638

[37] Chang Y, Nadigel J, Boulais N, Bourbeau J, Maltais F et al. (2011) CD8 positive T cells express IL-17 in patients with chronic obstructive pulmonary disease. Respir Res 12: 43. doi:10.1186/1465-9921-12-43. PubMed: 21477350. 21477350 PMC3082241

[38] Roark CL, Simonian PL, Fontenot AP, Born WK, O'Brien RL (2008) gammadelta T cells: an important source of IL-17. Curr Opin Immunol 20: 353-357. doi:10.1016/j.coi.2008.03.006. PubMed: 18439808. 18439808 PMC2601685

[39] Hitchon CA, El-Gabalawy HS (2011) Infection and rheumatoid arthritis: still an open question. Curr Opin Rheumatol 23: 352-357. doi:10.1097/BOR.0b013e3283477b7b. PubMed: 21532483. 21532483

[40] Suurmond J, Dorjée AL, Boon MR, Knol EF, Huizinga TW et al. (2011) Mast cells are the main interleukin 17-positive cells in anticitrullinated protein antibody-positive and -negative rheumatoid arthritis and osteoarthritis synovium. Arthritis Res Ther 13: R150. doi:10.1186/ar3466. PubMed: 21933391. 21933391 PMC3308080

[41] Chen D-Y, Chen Y-M, Chen H-H, Hsieh C-W, Lin C-C et al. (2011) Increasing levels of circulating Th17 cells and interleukin-17 in rheumatoid arthritis patients with an inadequate response to anti-TNF-α therapy. Arthritis Res Ther 13: R126. doi:10.1186/ar3431. PubMed: 21801431. 21801431 PMC3239366

[42] Aerts NE, De Knop KJ, Leysen J, Ebo DG, Bridts CH et al. (2010) Increased IL-17 production by peripheral T helper cells after tumour necrosis factor blockade in rheumatoid arthritis is accompanied by inhibition of migration-associated chemokine receptor expression. Rheumatology (Oxford) 49: 2264-2272. doi:10.1093/rheumatology/keq224. PubMed: 20724433. 20724433

[43] Chakir J, Shannon J, Molet S, Fukakusa M, Elias J et al. (2003) Airway remodeling-associated mediators in moderate to severe asthma: effect of steroids on TGF-beta, IL-11, IL-17, and type I and type III collagen expression. J Allergy Clin Immunol 111: 1293-1298. doi:10.1067/mai.2003.1557. PubMed: 12789232. 12789232

[44] Liu X, Yang P, Lin X, Ren X, Zhou H et al. (2009) Inhibitory effect of Cyclosporin A and corticosteroids on the production of IFN-gamma and IL-17 by T cells in Vogt-Koyanagi-Harada syndrome. Clin Immunol 131: 333-342. doi:10.1016/j.clim.2008.12.007. PubMed: 19200788. 19200788

[45] Miranda-Carús ME, Benito-Miguel M, Balsa A, Cobo-Ibáñez T, Pérez de Ayala C et al. (2006) Peripheral blood T lymphocytes from patients with early rheumatoid arthritis express RANKL and interleukin-15 on the cell surface and promote osteoclastogenesis in autologous monocytes. Arthritis Rheum 54: 1151-1164. doi:10.1002/art.21731. PubMed: 16575870. 16575870

[46] Marston B, Palanichamy A, Anolik JH (2010) B cells in the pathogenesis and treatment of rheumatoid arthritis. Curr Opin Rheumatol 22 %W http://view.ncbi.nlm.nih.gov/pubmed/20090526 : 307-315 10.1097/BOR.0b013e3283369cb8 PMC2947313 20090526

[47] Takemura S, Klimiuk PA, Braun A, Goronzy JJ, Weyand CM (2001) T cell activation in rheumatoid synovium is B cell dependent. J Immunol 167: 4710-4718. PubMed: 11591802. 11591802 10.4049/jimmunol.167.8.4710

[48] O'Neill SK, Shlomchik MJ, Glant TT, Cao Y, Doodes PD et al. (2005) Antigen-specific B cells are required as APCs and autoantibody-producing cells for induction of severe autoimmune arthritis. J Immunol 174: 3781-3788. PubMed: 15749919. 15749919 10.4049/jimmunol.174.6.3781

[49] Gavanescu I, Benoist C, Mathis D (2008) B cells are required for Aire-deficient mice to develop multi-organ autoinflammation: A therapeutic approach for APECED patients. Proc Natl Acad Sci U S A 105: 13009-13014. doi:10.1073/pnas.0806874105. PubMed: 18755889. 18755889 PMC2529049

[50] Wilson CL, Hine DW, Pradipta A, Pearson JP, van Eden W et al. (2012) Presentation of the candidate rheumatoid arthritis autoantigen aggrecan by antigen-specific B cells induces enhanced CD4(+) T helper type 1 subset differentiation. Immunology 135: 344-354. doi:10.1111/j.1365-2567.2011.03548.x. PubMed: 22182481. 22182481 PMC3372749

[51] Duddy ME, Alter A, Bar-Or A (2004) Distinct profiles of human B cell effector cytokines: a role in immune regulation? J Immunol 172: 3422-3427. PubMed: 15004141. 15004141 10.4049/jimmunol.172.6.3422

[52] Capolunghi F, Cascioli S, Giorda E, Rosado MM, Plebani A et al. (2008) CpG drives human transitional B cells to terminal differentiation and production of natural antibodies. J Immunol 180: 800-808. PubMed: 18178818. 18178818 10.4049/jimmunol.180.2.800

[53] Mauri C, Bosma A (2012) Immune regulatory function of B cells. Annu Rev Immunol 30: 221-241. doi:10.1146/annurev-immunol-020711-074934. PubMed: 22224776. 22224776

[54] Flores-Borja F, Bosma A, Ng D, Reddy V, Ehrenstein MR et al. (2013) CD19+CD24hiCD38hi B cells maintain regulatory T cells while limiting TH1 and TH17 differentiation. Sci Transl Med 5: 173ra123 PubMed: 23427243. 10.1126/scitranslmed.3005407 23427243

[55] Souto-Carneiro MM, Mahadevan V, Takada K, Fritsch-Stork R, Nanki T et al. (2009) Alterations in peripheral blood memory B cells in patients with active rheumatoid arthritis are dependent on the action of tumour necrosis factor. Arthritis Res Ther 11: R84. doi:10.1186/ar2718. PubMed: 19500335. 19500335 PMC2714135

[56] León B, Lund FE (2013) IL-17-producing B cells combat parasites. Nat Immunol 14: 419-421. doi:10.1038/ni.2593. PubMed: 23598388. 23598388

[57] Szodoray P, Jonsson R (2005) The BAFF/APRIL system in systemic autoimmune diseases with a special emphasis on Sjogren's syndrome. Scand J Immunol 62: 421-428. doi:10.1111/j.1365-3083.2005.01688.x. PubMed: 16305638. 16305638

[58] Mariette X, Roux S, Zhang J, Bengoufa D, Lavie F et al. (2003) The level of BLyS (BAFF) correlates with the titre of autoantibodies in human Sjogren's syndrome. Ann Rheum Dis 62: 168-171. doi:10.1136/ard.62.2.168. PubMed: 12525388. 12525388 PMC1754442

[59] Bosello S, Youinou P, Daridon C, Tolusso B, Bendaoud B et al. (2008) Concentrations of BAFF correlate with autoantibody levels, clinical disease activity, and response to treatment in early rheumatoid arthritis. J Rheumatol 35: 1256-1264. PubMed: 18528969. 18528969

[60] Genovese MC, Fleischmann RM, Greenwald M, Satterwhite J, Veenhuizen M et al. (2013) Tabalumab, an anti-BAFF monoclonal antibody, in patients with active rheumatoid arthritis with an inadequate response to TNF inhibitors. Ann Rheum Dis 72: 1461-1468. doi:10.1136/annrheumdis-2012-202775. PubMed: 23268367. 23268367

[61] Kwan Lam Lai, King Hung Ko O, Zheng BJ, Lu L (2008) Local BAFF gene silencing suppresses Th17-cell generation and ameliorates autoimmune arthritis. Proc Natl Acad Sci U S A 105: 14993-14998. doi:10.1073/pnas.0806044105. PubMed: 18820032. 18820032 PMC2567481

[62] Doreau A, Belot A, Bastid J, Riche B, Trescol-Biemont MC et al. (2009) Interleukin 17 acts in synergy with B cell-activating factor to influence B cell biology and the pathophysiology of systemic lupus erythematosus. Nat Immunol 10: 778-785. doi:10.1038/nrm2786. PubMed: 19483719. 19483719

[63] Korhonen R, Moilanen E (2010) Anti-CD20 antibody rituximab in the treatment of rheumatoid arthritis. Basic Clin Pharmacol Toxicol 106: 13-21. PubMed: 19686542. 19686542 10.1111/j.1742-7843.2009.00452.x

